# The effect of metformin on the pharmacokinetics of rifampicin, isoniazid, and pyrazinamide in adults with tuberculosis

**DOI:** 10.1128/aac.00748-26

**Published:** 2026-08-06

**Authors:** Jacques Rossouw, Robert Wallis, Hardy Kornfeld, Amit Singhal, Gary Maartens, Lubbe Wiesner, Paolo Denti, Roeland E. Wasmann

**Affiliations:** 1 Division of Clinical Pharmacology, Department of Medicine, University of Cape Town 37716, Cape Town, South Africa; 2 The Aurum Institute 72030, Johannesburg, South Africa; 3 Department of Medicine, UMass Chan Medical School 12262, Worcester, Massachusetts, USA; 4 A*STAR Infectious Diseases Labs, Agency for Science Technology and Research (A*STAR) 54759, Singapore, Republic of Singapore; Queen Mary University of London, London, United Kingdom

**Keywords:** *Mycobacterium tuberculosis*, first-line TB treatment, drug–drug interaction, population pharmacokinetic modeling, pharmacometrics

## Abstract

Metformin is under investigation as adjunctive host-directed therapy for tuberculosis (TB), which might interact with first-line TB treatment. We used population pharmacokinetic modeling to assess whether metformin alters first-line TB-drug pharmacokinetics in HIV/TB-coinfected adults without diabetes. Rifampicin, isoniazid, and pyrazinamide pharmacokinetics were investigated in participants from a randomized clinical trial of adjunctive metformin (500 mg twice daily to week 12) in adults starting HIV-associated TB treatment. Antiretroviral therapy (ART)-naïve participants initiated dolutegravir-based ART within 8 weeks. Intensive and semi-intensive sampling was conducted at week 5; concentration–time data were analyzed using non-linear mixed-effects modeling. Data from 78 individuals (43 receiving metformin, median weight 60.8 kg, 62.8% male, 79.5% on ART) were analyzed. Rifampicin and pyrazinamide were described by one-compartment models with linear elimination; typical clearances were 15.8 L/h (95% CI: 13.5–18.7) and 3.88 L/h (95% CI: 3.54–4.08), respectively. Isoniazid followed a two-compartment model with a mixture model for acetylator status; clearance was 10.5 L/h (95% CI: 9.44–11.9) in slow acetylators and 28.8 L/h (95% CI: 25.6–31.0) in fast/intermediate acetylators. Metformin reduced isoniazid bioavailability by 15.6% (95% CI: 4.45–26.4%, *P* < 0.009) and rifampicin bioavailability by 24.0% (95% CI: 7.83–36.3%, *P* < 0.007), decreasing the area under the curve from 0 to 24 h from 18.2 to 15.6 mg·h/L and from 35.9 to 27.1 mg·h/L, respectively. No significant effect on pyrazinamide was detected. We found that non-diabetic patients on metformin had lower isoniazid and rifampicin bioavailability. Lowered rifampicin exposure might be clinically relevant; simulations suggest that this could be offset by a single 150 mg rifampicin dose.

**CLINICAL TRIALS:**

This study is registered with ClinicalTrials.gov as NCT04930744.

## INTRODUCTION

Tuberculosis (TB) remains a leading cause of morbidity and mortality in low- and middle-income countries, particularly in those with high rates of HIV-associated TB (1, 2). A pooled meta-analysis of studies over the past three decades revealed that prevalence doubled from 7% to 14% between 1990 and 2022, with low- and middle-income countries disproportionately affected (3). HIV infection and diabetes mellitus (DM) increase susceptibility to TB and accelerate progression to active disease, resulting in large patient populations requiring prolonged and concurrent pharmacotherapy (4, 5).

People living with diabetes have an approximately threefold higher risk of developing active TB, increasing the likelihood of concurrent administration of both glucose-lowering therapy and first-line TB treatment (5, 6). The growing overlap of TB, HIV, and diabetes represents an emerging syndemic characterized by chronic comorbidity and polypharmacy (7). While drug–drug interactions between first-line TB drugs and antiretroviral therapy (ART) are well characterized and inform current clinical guidelines, substantially less is known about interactions between TB therapy and glucose-lowering agents (8, 9).

Metformin is classified by the World Health Organization as a first-line standard-of-care therapy for type 2 diabetes mellitus (10, 11), making coadministration with antitubercular treatment common in settings with a high burden of TB, HIV, and diabetes. In addition to its widespread use as a glucose-lowering agent, metformin is being investigated as a potential adjunctive therapy for TB, following reports that it exerts immunomodulatory effects, such as enhancing phagocytosis and reactive oxygen species production, which may influence host control of *Mycobacterium tuberculosis*-mediated inflammatory effects (12, 13). Immunomodulatory host-directed therapy may be particularly valuable throughout TB treatment, as modulation of inflammation during active therapy has the potential to limit the development of structural lung damage, including necrosis, cavitation, and fibrosis, thereby improving respiratory outcomes that persist beyond completion of curative treatment. These individuals have higher mortality rates compared to the general population, underscoring the need for additional interventions beyond first-line TB chemotherapy (14). The dual role of metformin as chronic therapy for diabetes and as a candidate host-directed therapy for TB further increases the probability of coexposure with first-line anti-TB drugs. Despite this, data describing the pharmacokinetic interactions between metformin and first-line TB drugs remain limited, particularly in populations where HIV infection and TB therapy may independently alter drug disposition (12, 15).

Prior investigations have largely focused on rifampicin’s effects on metformin pharmacokinetics, with limited evaluation of potential reciprocal effects on isoniazid, rifampicin, or pyrazinamide exposure (16). Notably, several studies have reported lower exposure to first-line TB drugs in patients with diabetes, suggesting that altered physiology associated with diabetes may influence drug pharmacokinetics (17).

Although these studies compared TB drug exposure between diabetic and non-diabetic individuals, interpretation remains challenging. While some studies reported on metformin use, they did not stratify pharmacokinetic outcomes by metformin exposure within the diabetic group. As a result, metformin use was variably present but not analytically separated, limiting the ability to distinguish the independent effects of diabetes from those of concomitant metformin therapy. This lack of stratification introduces confounding, as metformin use is closely correlated with diabetes status but not accounted for in the analysis. Consequently, it remains unclear whether the observed lowered TB drug concentrations are attributable to diabetes itself, metformin coadministration, or a combination of both.

A recent study by Kumar et al. attempted to address this uncertainty and reported lowered TB drug exposure in non-diabetic patients receiving metformin compared to those not receiving it, suggesting possible involvement of drug transporters (15). However, this study employed a non-compartmental analysis approach and was not designed to elucidate underlying mechanisms or disentangle the covariate and interindividual effects from those of metformin. It is therefore not yet fully clear whether metformin may alter the pharmacokinetics of first-line TB drugs and whether dosing adjustments may be necessary. This is a critical knowledge gap, given how often these treatments are administered concomitantly.

In this context, we evaluated whether coadministration of metformin is associated with clinically relevant changes in the pharmacokinetics of isoniazid, rifampicin, and pyrazinamide in HIV/TB-coinfected adults without diabetes using population pharmacokinetic modeling.

## MATERIALS AND METHODS

### Study design and participants

This pharmacokinetic substudy was nested within the METHOD trial, a phase IIa, randomized, open-label clinical trial evaluating the safety and tolerability of adding metformin to standard TB therapy and ART in adults with HIV-associated pulmonary TB.

Eligible participants were 18–65 years old with confirmed HIV-1 infection, radiological features of pulmonary TB, and microbiological confirmation via Xpert MTB/RIF (with a cycle threshold below 25) or culture, with rifampicin susceptibility documented. Key exclusions included diabetes or fasting glucose ≥7.0 mmol/L, metformin use within the preceding year, body mass index <17 kg/m², CD4 <50 cells/mm³, estimated glomerular filtration rate <60 mL/min/1.73 m², and AST/ALT ≥3 × ULN. The trial was conducted between August 2021 and November 2025 at two clinical research sites in South Africa: the Thembisa Clinical Research Center in Johannesburg and the Isango Lethemba TB Research Unit in Gqeberha.

Participants were randomized in a 1:1 ratio to either the control or the intervention arm. Participants in the intervention arm received metformin hydrochloride 500 mg (Mylan Pharmaceuticals) once daily for the first week and then twice daily for the remainder of the 12 weeks. All participants received once-daily dosing of a fixed-dose combination tablet containing isoniazid, rifampicin, pyrazinamide, and ethambutol (75/150/400/275 mg, Rifafour E-275; Sanofi-Aventis) based on body weight, according to the weight-based dosing of the South African national TB management guideline (18). Participants received dolutegravir-based ART during the study. ART-experienced participants were switched to dolutegravir twice daily at the start of TB treatment, while ART-naïve participants-initiated ART within 8 weeks of starting TB treatment, in accordance with national guidelines (19).

### Pharmacokinetic sampling

Participants were assigned to an intensive (predose and 1, 2, 4, 6, 8, and 10 h post-dose) or a semi-intensive (predose and 2 and 6 hours post-dose) pharmacokinetic visit at week 5. Participants were requested to fast overnight, and a standardized meal was provided 1 h after the observed dose. Blood samples were processed immediately and stored at −80°C. Isoniazid, rifampicin, and pyrazinamide plasma concentrations were quantified using validated LC–MS/MS methods at the University of Cape Town, with lower limits of quantification (LLOQ) of 0.117, 0.105, and 0.200 mg/L, respectively. Accuracy and precision across low-, medium-, and high-quality-control levels were 91.0%–105.1% and 2.7%–7.8% (relative standard deviation), respectively.

### Pharmacokinetic modeling

Non-linear mixed-effect modeling was performed in NONMEM 7.5.1 using first-order conditional estimation with eta-epsilon interaction (FOCE-I). PsN (version 4.6.0) was used to aid model-building procedures. Model development was tracked in Pirana (version 3.0.0), and data manipulation and visualization were conducted in R (version 3.6.0).

One- or two-compartment models with first-order absorption (with or without lag time or transit compartments) and first-order elimination were investigated, with structural model selection based on a significance threshold of *P* < 0.05. For isoniazid, a well-stirred liver model was explored to account for hepatic metabolism, with hepatic blood flow (*Q*_h_) fixed to 90 L/h for a standard 70 kg male and scaled allometrically and unbound fraction at 95%, as described previously (20, 21). Parameters were estimated relative to prehepatic bioavailability, with its typical value fixed at 1. For rifampicin, model development was informed by the previously published Chirehwa et al. framework (22), which guided the evaluation of alternative structural components, including saturable hepatic elimination, first-pass metabolism, and transit-compartment absorption models.

Random effects were incorporated assuming a log-normal distribution. Between-subject variability was evaluated for disposition parameters, while between-occasion variability was assessed for absorption parameters. Each occasion was defined as a dose together with the observations that followed it. Lower limit of quantification data were handled using the M7+ method (23). Residual error was described using combined additive and proportional components, with additive error bounded to be at least 20% of the LLOQ.

Body-size effects were incorporated using either weight or fat-free mass (24) with fixed allometric exponents (0.75 for clearances and 1 for volumes). The expected multimodality in isoniazid clearance due to NAT2 acetylator phenotype was accounted for using a mixture model, since genotype data were not available. Proportions of acetylator status (fast, intermediate, or slow) were estimated.

Following the establishment of the basic structural model, a systematic covariate analysis was performed using a stepwise forward inclusion and backward elimination approach. A significance level of *P* = 0.05 (ΔOFV ≥ 3.84, df = 1) was used for inclusion of covariates, while *P* = 0.01 (ΔOFV ≥ 6.63, df = 1) was required for retention during backward elimination. Covariate selection was also guided by physiological plausibility, improvements in diagnostic performance, and reduction in unexplained variability. The effects of metformin, concomitant ART use (not on ART, recently initiated on ART prior to the week 5 pharmacokinetic visit, or ART-experienced at study entry), age, alcohol use, or liver function markers were tested on pharmacokinetic parameters.

Model performance was assessed via goodness-of-fit plots and visual predictive checks (*n* = 1,000) throughout the modeling process. Parameter precision and 95% confidence intervals were obtained using sampling importance resampling.

### Simulations

Post hoc, individual parameter estimates from the final population pharmacokinetic models were used to derive individual steady-state area under the curve from 0 to 24 h (AUC_0–24h_) values. Simulations were performed in a virtual population of individuals generated from the NHANES database. The observed adult cohort was used to define a target body-size distribution, and NHANES 2017–2018 adults were resampled with replacement using density-based weighting derived from two-dimensional kernel density estimation, such that their joint weight–height distributions (with light stabilization on fat-free mass) matched those of the METHOD cohort. To isolate treatment effects, each virtual individual was replicated across scenarios and assigned to both metformin and no-metformin arms.

As no universally accepted pharmacokinetic target exists for first-line TB drugs, reference exposure ranges were derived from published adult studies using standard dosing and used as historical comparators (25, 26). For isoniazid, rifampicin, and pyrazinamide, these ranges were defined by the lowest and highest reported median steady-state AUC_0–24h_ values in the literature: 11.6–26.3 mg·h/L, 20.2–72.9 mg·h/L, and 233–429 mg·h/L, respectively (25, 26).

## RESULTS

### Study data

A total of 78 participants (62.8% male) were included in this analysis, of which 43 were on metformin. Semi-intensive visits were completed by 34 participants receiving metformin and 25 not receiving metformin, while intensive visits included 9 and 10 participants in the metformin and no-metformin arms, respectively. The median weight, fat-free mass, and age were 60.8 kg, 46.7 kg, and 43 years, respectively (Table 1). For isoniazid, rifampicin, and pyrazinamide, 68 (21.5%), 72 (23.2%), and 5 (1.6%) plasma observations were below the limit of quantification, respectively, the majority observed just prior to the directly observed dose. A total of 948 samples were included in this analysis. At the time of the week 5 pharmacokinetic visit, 62 participants (79.5%) were receiving ART, with the majority receiving tenofovir disoproxil fumarate–lamivudine–dolutegravir (*n* = 59) fixed-dose combination.

**TABLE 1:** Participant characteristics at the time of pharmacokinetic sampling^*a*^

Characteristics	No metformin (*N* = 37)	Metformin (*N* = 41)	Overall (*N* = 78)
No. of males	25 (68)	24 (59)	49 (63)
Age (years)	42 (37–49)	43 (35–47)	43 (35–49)
Weight (kg)	62.1 (57.0–70.3)	57.8 (52.2–64.7)	60.8 (53.9–67.8)
Height (cm)	168 (163–175)	165 (159–171)	166 (160–172)
Fat-free mass (kg)^*b*^	47.6 (43.6–53.0)	45.2 (40.3–50.5)	46.7 (41.1–51.8)
Body mass index (kg/m^2^)	22.3 (20.0–25.2)	21.6 (19.0–23.3)	21.8 (19.6–24.0)
Alcohol consumption (>5 g/day)	16 (43.2)	11 (26.8)	27 (34.6)
Creatinine clearance (mL/min)^*c*^	104 (85.0–130.0)	111 (86.6–122.0)	110 (86.6–123.0)
Bilirubin (µmol/L)	6.0 (4.0–7.0)	5.5 (4.0–7.0)	5.5 (4.0–7.0)
Aspartate aminotransferase (U/L)	31.0 (25.0–38.0)	31.5 (26.0–38.0)	31.2 (25.1–38.0)
Alanine aminotransferase (U/L)	20.0 (16.5–28.5)	20.0 (16.0–27.0)	20.0 (16.1–27.9)
Antiretroviral therapy			
Efavirenz–emtricitabine–tenofovir	1 (2.9)	0 (0)	1 (1.3)
Abacavir–lamivudine–dolutegravir	1 (2.9)	1 (2.4)	2 (2.6)
Tenofovir–lamivudine–dolutegravir	30 (86)	29 (69)	59 (77)
ART status at week 5 PK visit			
Not on any ART	5 (13.5)	11 (26.8)	16 (20.5)
Recently initiated (day 0 to week 5 PK visit)	11 (29.7)	12 (29.3)	23 (29.5)
ART experienced (on ART before enrollment)	21 (56.8)	18 (43.9)	39 (50)

^*a*^ Data are presented as median (IQR) or *n* (%).

^*b*^ Fat-free mass was calculated according to Janmahasatian et al. (24).

^*c*^ Creatinine clearance was calculated using the Cockcroft–Gault method (27).

### Population pharmacokinetic models

Isoniazid pharmacokinetics was best described by a two-compartment model (ΔOFV = −29.2, *P* < 0.001, compared to the one-compartment model) with first-order absorption and elimination via hepatic extraction (with first-pass effect). The inclusion of hepatic first-pass improved the model (ΔOFV = −9.32) over standard elimination from the central compartment. A mixture model was included to account for NAT2 phenotype (ΔOFV = −34.2, *P* < 0.001), and it reduced the unexplained between-subject variability in clearance from 54.6% to 17% by allocating 69.5% to fast/intermediate and ~30.5% to slow acetylators. Further stratification into a third category (splitting fast and intermediate) was not supported by our data.

Allometric scaling with FFM on disposition parameters (including hepatic blood flow *Q*_h_) yielded a better fit compared to the total body weight (dOFV = −10.7). For a typical study participant with an FFM of 45 kg, clearance was estimated to be 28.8 L/h for fast/intermediate acetylators and 10.5 L/h for slow acetylators. Participants on metformin had a 15.6% (95% CI: 4.45–26.4) lower bioavailability compared to those not on metformin (ΔOFV = −6.76, *P* < 0.01). This is also reflected in the lower exposure metrics observed in participants on metformin (see Table 2), with corresponding decreases in AUC_0–24h_.

**TABLE 2:** Model-derived secondary pharmacokinetic parameters of isoniazid, rifampicin, and pyrazinamide with and without metformin coadministration*^a^*^,^^*b*^

		No metformin		Metformin	
		AUC_0–24h_ (mg·h/L)	*C*_max_ (mg/L)	AUC_0–24h_ (mg·h/L)	*C*_max_ (mg/L)
Isoniazid	Fast/intermediate acetylators	11.0 (28.7)	2.96 (54.7)	9.43 (30.1)	3.00 (23.6)
	Slow acetylators	30.1 (28.6)	4.48 (24.4)	25.8 (29.5)	4.30 (37.6)
Rifampicin		35.9 (49.8)	6.64 (32.7)	27.1 (60.4)	5.06 (38.7)
Pyrazinamide		419 (31.7)	38.1 (24.9)	370 (36.4)	36.8 (23.8)

^*a*^ Values are presented as geometric mean with coefficient of variation (%).

^*b*^ Area under the curve and maximum concentration (*C*_max_).

Rifampicin pharmacokinetics was adequately described by a one-compartment disposition model with first-order absorption and elimination. Allometric scaling using FFM provided a better fit than total body weight (ΔOFV = −11.5). A more complex model incorporating saturable hepatic elimination did not significantly improve the fit. For a typical study participant, clearance was estimated to be 15.8 L/h. Incorporation of metformin treatment as a covariate on bioavailability significantly improved model fit (ΔOFV = −7.26, *P* < 0.01), and participants in the metformin arm had a 24.0% (95% CI: 7.83-36.3) lower rifampicin bioavailability. This corresponds to an estimated geometric mean AUC_0–24h_ (%CV) of approximately 27.1 mg·h/L (60.4%) in the metformin arm compared to 35.9 mg·h/L (49.8%) in the non-metformin arm.

Pyrazinamide plasma concentration data were best described by a one-compartmental disposition model with first-order absorption with four transit compartments (ΔOFV = −73.4, *P* < 0.0001 compared to a first order) and first-order elimination. Allometric scaling with FFM provided a better fit over total body weight (ΔOFV = −28.5). Clearance was estimated to be 3.88 L/h for a typical study participant (FFM = 45 kg). We did not find an effect of metformin on bioavailability. Inclusion of a metformin effect on the absorption rate improved model fit (ΔOFV = −6.12; *P* = 0.013), corresponding to a slower absorption. However, this effect was not retained in the final model because it did not meet the predefined retention criterion for backward elimination (*P* < 0.01).

**Fig 1:**
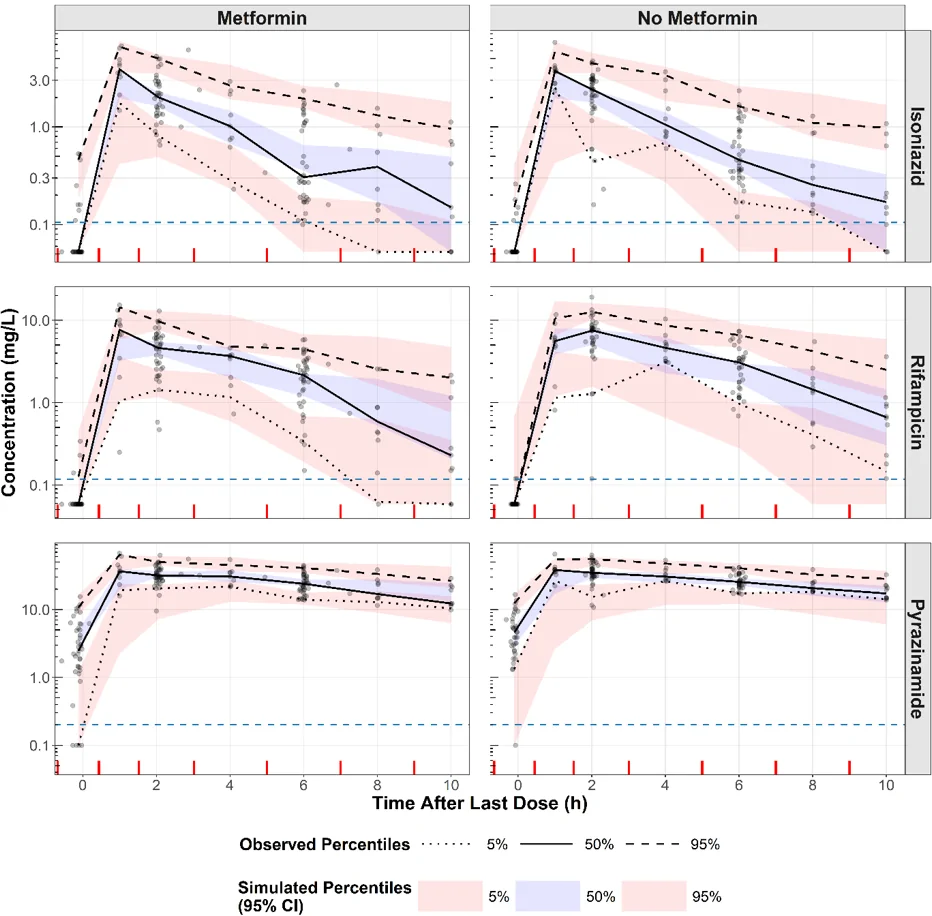
Visual predictive checks of steady-state plasma concentration–time profiles for isoniazid, rifampicin, and pyrazinamide stratified by metformin coadministration. Solid and dashed lines represent the 5th, 50th, and 95th percentiles of the observed data. The shaded regions represent the model-predicted 95% confidence intervals for the corresponding percentiles, derived from 1,000 simulated data sets. The red tick marks on the *x*-axis denote the bin edges. Points indicate observed concentrations, while concentrations below the lower limit of quantification (LLOQ) are shown as dashed blue lines (LLOQ/2) for both observed and simulated data for visualization purposes. Isoniazid, rifampicin, and pyrazinamide lower limits of quantification are 0.117, 0.105, and 0.200 mg/L, respectively.

All final models are presented in Table 3, and the NONMEM control stream is included in the Supplemental material. Visual predictive checks stratified by metformin coadministration are presented in Fig. 1 and show that the models provide an adequate description of the data. For isoniazid, a VPC stratified by predicted acetylator status also showed adequate model fit (Fig. S1).

**TABLE 3:** Final pharmacokinetic parameter estimates for isoniazid, rifampicin, and pyrazinamide

Typical values (95% confidence interval) (shrinkage)			
Parameters	Isoniazid	Rifampicin	Pyrazinamide
Clearance (L/h)^*a*^		15.8 (13.5–18.7)	3.88 (3.54–4.08)
Slow acetylator (L/h)*^a^*^*,**c*^	10.5 (8.97–11.3)		
Fast/intermediate acetylator (L/h)^*a**,**c*^	28.8 (24.3–29.5)		
Population proportion of NAT2 acetylator groups			
Slow acetylators	0.305 (0.226–0.435)		
Fast/intermediate acetylators	0.695 (0.565–0.774)		
Central volume of distribution (L)^*a*^	41.3 (37.7–45.0)	50.8 (42.9–60.4)	40.1 (37.7–41.6)
Intercompartmental clearance (L/h)^*a*^	2.97 (2.13–3.65)		
Peripheral volume of distribution (L)^*a*^	30.2 (22.1–43.1)		
Bioavailability	1 fixed	1 fixed	1 fixed
Number of absorption transit compartments			4 fixed
Mean transit time (h^−1^)			0.424 (0.0638–0.511)
First-order absorption rate constant (h^−1^)	3.14 (2.55–5.16)	1.03 (0.774–1.48)	3.45 (2.56–4.15)
Hepatic blood flow (L/h)^*a*^^,*b*^	76.3 fixed		
Metformin effect size (%)			
Bioavailability	−15.6 (−4.45 to −26.4)	−24.0 (−7.83 to −36.3)	
Between-subject variability (%)			
Clearance	16.9 (13.4–20.9) (4%)	42.2 (34.1–53.6) (9%)	31.4 (26.2–38.0) (10%)
Between-occasion variability (%)			
Bioavailability (*F*)	21.8 (13.3–33.6) (34%)	31.2 (22.4–42.7) (16%)	16.9 (13.5–21.2) (11%)
First-order absorption rate constant (Ka)	156 (155–598) (14%)	31.3 (6.93–56.6) (41%)	96.1 (78.3–348) (7%)
Mean transit time			267 (126–408) (7%)
Residual unexplained error			
Proportional error (%)	17.9 (15.7–21.2)	32.1 (29.0–37.8)	7.40 (5.72–8.90)
Additive error (mg/L)	0.02 fixed	0.02 fixed	0.935 (0.608–1.34)

^*a*^ All the disposition parameters were allometrically scaled. The reported typical values refer to the typical individual in the cohort with a fat-free mass of 45 kg.

^*b*^ Hepatic blood flow is described in the literature as 90 L/h for a standard 70 kg male with an FFM of 56 kg. The typical FFM in our cohort is 45 kg, and the hepatic blood flow was scaled accordingly.

^*c*^ The reported value is apparent clearance, which was calculated as CL_intrinsic_ × *F*_u_ (fraction unbound = 0.95).

### Simulations

**Fig 2:**
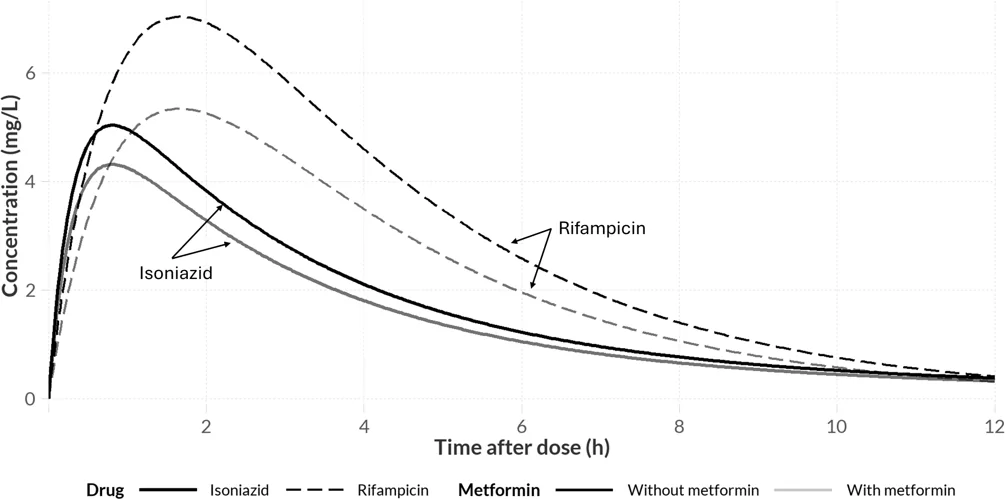
Simulated steady-state rifampicin and isoniazid plasma concentration–time profiles for a typical individual (fat-free mass = 45 kg, slow acetylator status), showing model-predicted concentrations stratified by metformin coadministration. Simulated weight-based doses are 300 mg for isoniazid and 600 mg for rifampicin.

**Fig 3:**
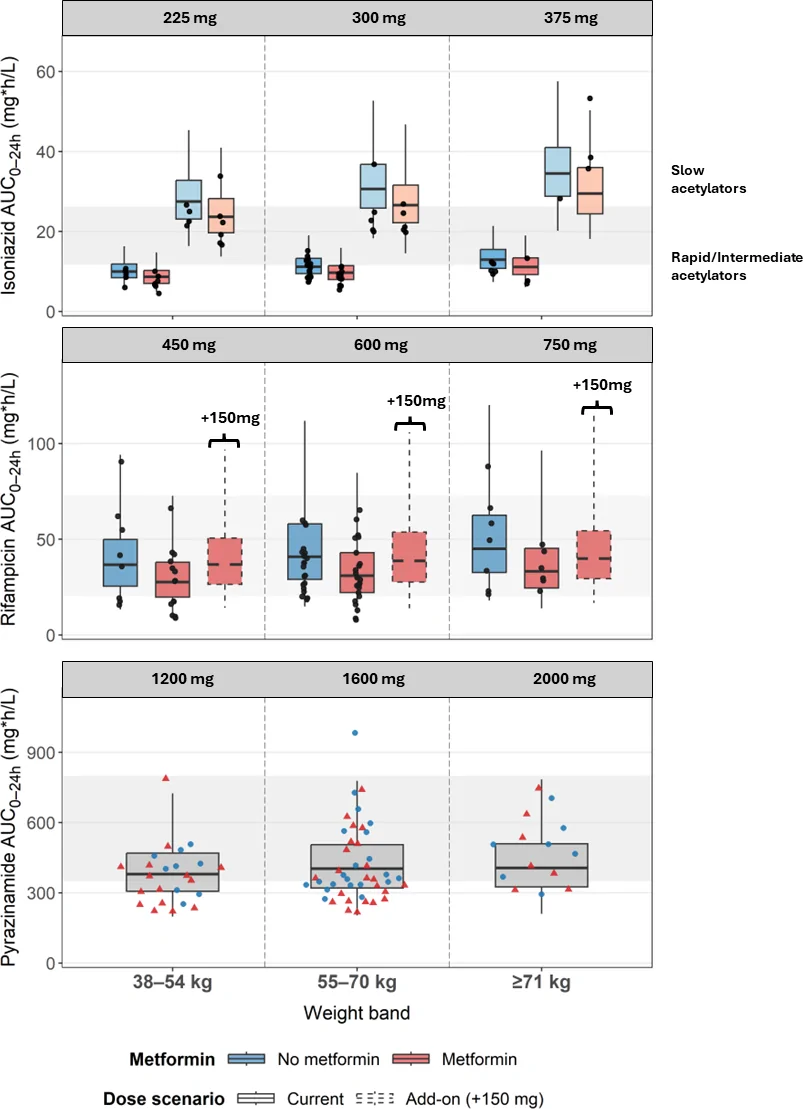
Model-predicted AUC_0–24h_ distributions for isoniazid (top), rifampicin (middle), and pyrazinamide (bottom) in a virtual cohort of 1,000 individuals. The dotted line box plot represents a simulated cohort that received an extra rifampicin dose (+150 mg). Boxes and whiskers show the median and 95% prediction interval; points represent observed participant AUC estimates; and gray-shaded bands indicate historical adult reference exposure ranges at standard dosing (25, 26). As no significant metformin effect was found for pyrazinamide, the box plot was not split between metformin vs no metformin.

Simulated steady-state plasma concentration–time profiles for rifampicin and isoniazid in a typical individual (FFM = 45 kg) are presented in Fig. 2. Steady-state plasma concentrations were simulated from standard rifampicin and isoniazid doses in each weight band and are depicted in Fig. 3. Importantly, the observed rifampicin drug–drug interaction was effectively mitigated through the coadministration of an additional 150 mg rifampicin dose.

## DISCUSSION

This study evaluated the effect of metformin coadministration on the pharmacokinetics of isoniazid, rifampicin, and pyrazinamide in adults with HIV and pulmonary TB, using population pharmacokinetic modeling to characterize exposure differences and to explore their clinical relevance. In our population pharmacokinetic models, metformin use was associated with lowered oral bioavailability of isoniazid (−15.6%) and rifampicin (−24.0%), resulting in correspondingly lower exposure, while no significant effect was found for pyrazinamide. Model-based simulations revealed that the median AUC_0–24h_ values of the drugs remained within the range of previously reported medians, although being at the lower end.

The observed lower isoniazid and rifampicin bioavailability is consistent with findings from the METRIF trial and related studies (12, 15). In the study by Padmapriydarsini et al., coadministration with metformin was associated with a lower rifampicin AUC_0–∞_ from 30.5 to 23.9 mg·h/L (21.6% lower) and an isoniazid AUC_0–∞_ from 30.4 to 20.8 mg·h/L (31.6% lower). Similarly, Kumar et al. reported lower rifampicin AUC_0–∞_ from 33.5 to 22.8 mg·h/L (31.9% lower) and isoniazid from 41.3 to 29.1 mg·h/L (29.5% lower) with metformin coadministration. These findings are in line with the lowered exposures observed in our study. Pyrazinamide showed a directional effect for slower absorption in our model, but this effect was not retained during backward elimination. Taken together, this pattern suggests a shared, non-specific mechanism rather than drug-specific interactions.

Metformin has been implicated in altered drug concentrations through multiple pathways, including effects on intestinal absorption (e.g., changes in gut motility or bile acid handling), indirect modulation of hepatic or biliary elimination, and broader alterations in the gut environment, including the intestinal microbiota (28–33). These gastrointestinal effects provide a plausible explanation for the observed changes in absorption kinetics of coadministered TB drugs, particularly because delays in rifampicin absorption were reported in diabetic populations before (34). Although saturation of first-pass metabolism has been reported for both rifampicin and isoniazid, potentially linking slower absorption to reduced bioavailability, our models did not support first-pass saturation and did not demonstrate a distinct delay in absorption separate from the observed changes in bioavailability (35, 36).

In parallel, metformin disposition itself is highly dependent on membrane transporters. As a hydrophilic cation, it relies on organic cation transporters and multidrug and toxin extrusion proteins (MATEs) for absorption, distribution, and elimination (37). Evidence from clinical interaction studies with rifampicin, a potent inducer of drug-metabolizing enzymes and transporters, demonstrates increased metformin exposure, underscoring the importance of transporter-mediated processes (16). Furthermore, Kumar et al. reported downregulation of MATE1 expression in patients receiving metformin, accompanied by lowered exposure to first-line TB drugs, suggesting that metformin may also modulate transporter activity (15). Collectively, these findings support the biological plausibility of bidirectional pharmacokinetic interactions between metformin and first-line TB therapy, with metformin affecting TB drug exposure (potentially via gastrointestinal mechanisms) and rifampicin influencing metformin disposition through transporter induction.

Multiple studies have evaluated plasma concentrations of first-line anti-TB drugs in patients with diabetes, yet their findings remain inconsistent, despite evidence that diabetes negatively affects TB treatment outcomes (17, 38–43). Some reports describe 25%–53% lower rifampicin exposure in patients with diabetes, which is consistent with the decrease we observed in the metformin arm of our study (44). Conversely, others report no meaningful differences compared with non-diabetic individuals (17, 38, 39, 42), but the absence of interaction may be attributable to metformin not being administered concurrently with TB treatment. A systematic review and meta-analysis did, however, find absorption of rifampicin was altered in diabetic populations across studies (17). The discrepancies in plasma concentrations between diabetics and non-diabetics have been previously attributed to higher body weight in patients with diabetes, thus leading to recommendations for dose adjustments in heavier individuals (42). However, because our model accounts for body weight through allometric scaling, we can distinguish weight-related effects from other sources of variability. In addition, most previous studies have relied on non-compartmental analysis to compare separate diabetes and non-diabetes groups, an approach that limits mechanistic insight and handling of confounders (e.g., dose and body size) in the characterization of interindividual variability. Taken together, our findings in a non-diabetic population suggest metformin-mediated processes are a major driver for lowered exposures.

In the present analysis, rifampicin and isoniazid exposures with metformin coadministration remained within previously reported reference ranges, albeit toward the lower end of those distributions (25, 26, 45). This observation warrants consideration of rifampicin dose adjustment, given the sensitivity of *M. tuberculosis* to rifampicin exposure levels (46). Metformin is increasingly recognized for its host-directed and immunomodulatory effects in TB, although the clinical relevance of these effects remains to be fully established. This may warrant evaluation of simple dosing adaptations that preserve these potential benefits while offsetting lowered rifampicin exposure. A pragmatic approach could include an additional rifampicin dose (i.e., 150 mg capsule), particularly given the exposure differences observed here, which are comparable in magnitude to those reported elsewhere (44, 47–49). In parallel, there is increasing interest in the use of higher rifampicin doses to improve drug exposure and pharmacodynamic target attainment, supported by multiple clinical and pharmacokinetic studies demonstrating enhanced bactericidal activity and faster culture conversion at doses above the historical standard (e.g., 20–35 mg/kg vs the standard 10 mg/kg) (50–52). Within this evolving framework of rifampicin dose optimization, a dose increment such as 150 mg would remain well within the range of doses currently under active investigation and may represent a simple strategy to mitigate lower exposures without compromising the adjunctive benefits of metformin (14, 53).

When contextualized against established sources of exposure variability, the lower isoniazid exposure observed with metformin coadministration can be considered modest, especially considering that NAT2 acetylator status remains the dominant determinant of isoniazid pharmacokinetics, with fast acetylators exhibiting a substantially lower AUC_0–24h_ compared to the slow and intermediate counterparts. This effect exceeds the relatively modest magnitude of the metformin-associated bioavailability effect observed in this study. Consequently, although the metformin effect was statistically detectable, it is small relative to the inherent interindividual variability driven by acetylation phenotype.

When considered alongside the rifampicin results, these findings suggest a broadly similar effect of metformin across the three orally administered first-line TB drugs. Lowered exposure was observed for rifampicin and isoniazid, whereas pyrazinamide showed only a modest change in absorption rate without a corresponding decrease in overall exposure, possibly reflecting its high intrinsic absorptive capacity. This differential behavior is consistent with known differences in oral absorption characteristics among these agents, with rifampicin and isoniazid exhibiting more variable and limited absorption, while pyrazinamide is rapidly and efficiently absorbed across a wide dose range (54, 55). Accordingly, metformin-related alterations in gastrointestinal physiology and potentially transporter-mediated processes may preferentially affect drugs with more complex absorption profiles. As all three drugs were administered within the same fixed-dose combination, these findings are unlikely to reflect differences in dosing conditions and instead suggest a shared effect on absorption.

This study has limitations. Although this was a randomized parallel-arm study, allowing balanced comparison between groups, it does not permit within-subject assessment of drug exposure with and without metformin. A crossover design would offer greater control of interindividual variability and strengthen causal inference and should be considered in future studies. Another limitation is that our trial used relatively low metformin doses, administered twice daily, to minimize gastrointestinal intolerance and align with existing guidance on coadministration with dolutegravir (56). Consequently, the extent to which the observed pharmacokinetic effects would generalize to standard or higher metformin dosing regimens remains uncertain, especially since the interaction might be in the gastrointestinal tract. If the metformin–antitubercular interaction is dose dependent, the magnitude of the effect may be underestimated in the present study. In addition, the first-line TB drugs were always administered concurrently with metformin. Administration of metformin separately in time would have helped distinguish time-dependent gastrointestinal or absorption-related interactions from systemic mechanisms and should be considered in future investigations.

Important questions remain regarding whether these exposure-level effects also influence treatment response beyond diabetes-related effects on treatment outcomes. Future research should prioritize crossover studies evaluating higher metformin doses, studies examining pharmacokinetic effects in specific disease states (e.g., DM), and investigations evaluating treatment outcomes.

### Conclusion

In conclusion, our population pharmacokinetic analysis revealed that metformin coadministration is associated with a lower oral bioavailability of rifampicin and isoniazid in adults with TB/HIV coinfection, while no effect on pyrazinamide exposure was found. Simulations show that this interaction can be offset by the addition of a single 150 mg dose during metformin coadministration.

## SUPPLEMENTAL MATERIAL

Supplemental material aac.00748-26-s0001.docx Fig. S1; model code and VPCs.

ASM does not own the copyrights to Supplemental Material that may be linked to, or accessed through, an article. The authors have granted ASM a non-exclusive, world-wide license to publish the Supplemental Material files. Please contact the corresponding author directly for reuse.
