# Supplementary material for: The effect of metformin on the pharmacokinetics of rifampicin, isoniazid, and pyrazinamide in adults with tuberculosis
Source: Antimicrob Agents Chemother. 2026 Aug 6;70(9):e00748-26. doi: 10.1128/aac.00748-26 (PMC13712770; doi:10.1128/aac.00748-26)
Supplement: Supplementary file 1 [file aac.00748-26-s0001.docx]

**SUPPLEMENTARY MATERIAL**

The effect of metformin on the pharmacokinetics of Rifampicin, Isoniazid, and Pyrazinamide in adults with Tuberculosis

Jacques Rossouw (1), Robert Wallis (2), Hardy Kornfeld (3), Amit Singhal (4), Gary Maartens (1), Lubbe Wiesner (1), Paolo Denti (1), Roeland E. Wasmann (1)

(1) Division of Clinical Pharmacology, Department of Medicine, University of Cape Town, Cape Town, South Africa.

(2) The Aurum Institute, Johannesburg, South Africa.

(3) Department of Medicine, UMass Chan Medical School, Worcester, MA 01655 USA.

(4) A*STAR Infectious Diseases Labs, Agency for Science Technology and Research (A*STAR), Singapore 138648, Republic of Singapore.

**Corresponding author:**

Jacques Rossouw

Division of Clinical Pharmacology

Department of Medicine

University of Cape Town

Cape Town, South Africa

rssmic032@myuct.ac.za

Table of Contents

[**S1. Visual predictive checks (VPC) for Isoniazid, stratified by acetylator status and sampling intensity (Semi-intensive vs Intensive).**	3](#_Toc230621285)

[**S2. Isoniazid final model NONMEM code**	4](#_Toc230621286)

[**S3. Rifampicin final model NONMEM code**	10](#_Toc230621287)

[**S4. Pyrazinamide final model NONMEM code**	14](#_Toc230621288)

**S1. Visual predictive checks (VPC) for Isoniazid, stratified by acetylator status.**

**Figure S1**:Visual predictive checks (VPCs) of steady-state plasma concentration–time profiles for isoniazid stratified by acetylator status. Solid and dashed lines represent the 5th, 50th, and 95th percentiles of the observed data. The shaded regions represent the model-predicted 95% confidence intervals for the corresponding percentiles, derived from 1,000 simulated datasets. Points indicate observed concentrations, while concentrations below the lower limit of quantification (LLOQ) are shown as dashed blue lines (LLOQ/2) for both observed and simulated data for visualization purposes. Isoniazid lower limits of quantification are 0.105.

# **S2. Isoniazid final model NONMEM control stream**

```
$PROBLEM    Method trial – isoniazid final model
$INPUT      ID AMT DV TIME EVID MDV OCC BLQ CENS FFM_KG
  METFORMIN NAT
$DATA       data.csv IGNORE=@
$ABBR DERIV2=NO
$SUBROUTINE ADVAN4 TRANS1
;-------------------------------------------------------------
$MIX
NSPOP=2               ; 2 POPULATIONS (SLOW AND FAST/INTERMEDIATE ACETYLATORS)
P(1)=THETA(10)         ; PROBABILITY OF BEING A FAST/INTERMEDIATE ACETYLATOR
P(2)=1-P(1)           ; PROBABILITY OF BEING A SLOW ACETYLATOR
;-------------------------------------------------------------
$PK
; ------- BSV
BSVCL   = ETA(1)
; ---------- BOV
BOVBIO = 0
IF (OCC.EQ.1)BOVBIO = ETA(2)
IF (OCC.EQ.2)BOVBIO = ETA(3)
BOVKA =  0
IF (OCC.EQ.1)BOVKA = ETA(4)
IF (OCC.EQ.2)BOVKA = ETA(5)
; -----------------------Allometric scaling ------------------
;   Body-size covariates
TVFFM = 45; 45.6kg in METHOD TRIAL
;--------- Allometric scaling and covariates
ALLMCL_FFM = (FFM_KG    / TVFFM)**0.75   ; CL ~ FFM^0.75
ALLMV_FFM  = (FFM_KG    / TVFFM)         ; V  ~ FFM^1.0

;--------- Allometric scaling for liver
ALLMCL_FFM_HEP = (FFM_KG/56.1)**0.75	; CL ~ FFM^0.75
ALLMV_FFM_HEP = (FFM_KG/56.1)			; V  ~ FFM^1.0
;---------Typical values--------------------------------------
;---------------------Mixture Model---------------------------
EST = MIXEST
IF(MIXNUM.EQ.1) IMP_NAT = 1 ; FAST/INTERMEDIATE ACETYLATORS
IF(MIXNUM.EQ.2) IMP_NAT = 2 ; SLOW ACETYLATORS

IF(IMP_NAT.EQ.1) THEN
      TVCL = THETA(1)*ALLMCL_FFM ; FAST/INTERMEDIATE ACETYLATORS
ENDIF

IF(IMP_NAT.EQ.2) THEN
      TVCL = THETA(2)*ALLMCL_FFM ; CL FOR SLOW ACETYLATORS
ENDIF

; -------- METFORMIN effect on BIO (multiplicative) ----------
MET_BIO = 1
IF(METFORMIN.EQ.1) MET_BIO = 1+THETA(13)
;------------------------------------------------------------
TVV   = THETA(3) * ALLMV_FFM
TVKA  = THETA(4)
TVBIO = THETA(5) * MET_BIO
TVV3  = THETA(8) * ALLMV_FFM
TVQ   = THETA(9) * ALLMCL_FFM
;--------------------------HEPATIC CL-------------------------
TVQH=THETA(11)*ALLMCL_FFM_HEP; PLASMA FLOW RATE
TVFU=THETA(12)               ; UNBOUND PLASMA FRACTION OF INH
;-----------Define parameters---------------------------------
CLINT  = TVCL*EXP(BSVCL) ; INTRINSIC CLEARANCE
V   = TVV ; CENTRAL VOL.
KA  = TVKA*EXP(BOVKA) ; ABS. RATE CONSTANT
BIO = TVBIO*EXP(BOVBIO) ; BIOAVAILABILITY
V3 = TVV3*; PERIPHERAL VOLUME
Q = TVQ; INTERCOMPARTMNTAL CL
QH = TVQH ; BLOOD FLOW TO THE LIVER
FU = TVFU ; FRACTION UNBOUND
;-------------------------------------------------------------
CL = CLINT ;

; re-parameterization
;--------  Transfer constants for liver model ---------
EH  = (CLINT*FU)/((CLINT*FU)+QH) ; fraction undergoing first pass extraction
FH  = 1 - EH ; fraction available after 1st pass to go to systemic circulation
CLH = QH*EH

K = CLH /V
K23 = Q/V   ; (rate constant from central to peripheral 1)
K32 = Q/V3  ;(rate constant from peripheral 1 to central)

F1 = FH*BIO
S2 = V

;============================================================;
$ERROR
IPRED=A(2)/V
LLOQ = 0.105 ;
CENS_THR = LLOQ
PROP = IPRED*THETA(6)
ADD = THETA(7)+(CENS_THR*0.2)

; BLQ handling method M7+, Wijk et al. CPT:PSP 2025 14(6)1042:1049
IF (ICALL/=4.AND.BLQ==1) ADD = ADD + LLOQ

W = SQRT(ADD**2+PROP**2)
IF (W.LE.0.000001) W=0.000001

IRES=DV-IPRED
IWRES=IRES/W

Y = IPRED + W*ERR(1)

;-------------------------------------------------------------
$THETA
(28.8) ; 1 CL [L/h] FAST/INTERMEDIATE ACETYLATORS
(10.5) ; 2 CL [L/h] SLOW ACETYLATORS
(41.3) ; 3 V [L]
(3.14) ; 4 KA [1/h]
(1) FIX ; 5 BIO
(0.179) ; 6 PROP []
(0) FIX ; 7 ADD [mg/L]
(30.2) ; 8 V3 [L]
(2.97) ; 9 Q [L/h]
(0.695) ; 10 PROPORTION WITH FAST/INTERMED CL
(90) FIX ; 11 QH (L/h) -> AVERAGE BLOODFLOW TO THE LIVER FOR A 70kg man (90 L/H) * (FFM AVERAGE OF METHOD COHORT/AVERAGE FFM OF A 70kg MAN)**0.75 = 76.3L/H
(0.95) FIX ; 12 FRACTION UNBOUND
(-0.143) ; 13 MET_BIO

$OMEGA BLOCK(1) 0.0282 ; 1 BSV CL
;-------------------------------------------------------------
$OMEGA BLOCK(1) 0.0464 ; 2 BOVBIO (OCC1)
$OMEGA  BLOCK(1) SAME  ; 3BOVBIO (OCC2)
;-------------------------------------------------------------
$OMEGA BLOCK(1) 1.24 ; 4 BOVKA (OCC1)
$OMEGA  BLOCK(1) SAME ; 5 BOVKA (OCC2)
;-------------------------------------------------------------
$SIGMA 1 FIX
;-------------------------------------------------------------
$ESTIMATION MAXEVAL=9999 METHOD=1 INTER
```

# **S3. Rifampicin final model NONMEM control stream**

```
$PROBLEM    Method trial – rifampicin final model
$INPUT      ID AMT DV TIME EVID MDV OCC BLQ FFM_KG
  METFORMIN
$DATA       data.csv IGNORE=@
$ABBR DERIV2=NO
$SUBROUTINE ADVAN2 TRANS1 ;
;-------------------------------------------------------------
$PK
; ------- BSV
BSVCL   = ETA(1)
; ---------- BOV
BOVBIO = 0
IF (OCC.EQ.1)BOVBIO = ETA(2)
IF (OCC.EQ.2)BOVBIO = ETA(3)
BOVKA =  0
IF (OCC.EQ.1)BOVKA = ETA(4)
IF (OCC.EQ.2)BOVKA = ETA(5)
;------------------- Allometric scaling ----------------------
TVFFM = 45; 45.6kg in METHOD TRIAL
;--------- Allometric scaling and covariates ----------------
ALLMCL_FFM = (FFM_KG    / TVFFM)**0.75   ; CL ~ FFM^0.75
ALLMV_FFM  = (FFM_KG    / TVFFM)         ; V  ~ FFM^1.0
;---------------------Adding covariates-----------------------
MET_BIO = 1
IF(METFORMIN.EQ.1) MET_BIO = (1+THETA(7))
;----------------------Typical values-------------------------
TVCL = THETA(1)*ALLMCL_FFM;
TVV = THETA(2)*ALLMV_FFM
TVKA = THETA(3);
TVBIO = THETA(4)*MET_BIO
;---------------------Define parameters-----------------------
CL  = TVCL*EXP(BSVCL) ; CLEARANCE ;
V   = TVV; CENTRAL VOL.
KA  = TVKA*EXP(BOVKA) ; ABS. RATE CONSTANT
BIO = TVBIO*EXP(BOVBIO) ; BIOAVAILABILITY
;-------------------------------------------------------------
; re-parameterization
K = CL/V ;(rate constant of elimination)
F1 = BIO
S2 = V

;-------------------------------------------------------------
$ERROR
IPRED=A(2)/V
LLOQ = 0.117 ;
CENS_THR = LLOQ
PROP = IPRED*THETA(5)
ADD = THETA(6)+(CENS_THR*0.2)

; BLQ handling method M7+, Wijk et al. CPT:PSP 2025 14(6)1042:1049
IF (ICALL/=4.AND.BLQ==1) ADD = ADD + LLOQ

W = SQRT(ADD**2+PROP**2)
IF (W.LE.0.000001) W=0.000001

IRES=DV-IPRED
IWRES=IRES/W

Y = IPRED + W*ERR(1)
;-------------------------------------------------------------
$THETA
(15.8) ; 1 CL [L/h]
(50.8) ; 2 V [L]
(1.03) ; 3 KA [1/h]
(1) FIX ; 4 BIO
(0.321) ; 5 PROP []
(0) FIX ; 6 ADD [mg/L]
(-0.240) ; 7 MET_BIO
;-------------------------------------------------------------
$OMEGA BLOCK(1) 0.164 ; 1 BSV CL
;-------------------------------------------------------------
$OMEGA BLOCK(1) 0.0930 ; 2 BOVBIO (OCC1)
$OMEGA  BLOCK(1) SAME  ; 3 BOVBIO (OCC2)
;-------------------------------------------------------------
$OMEGA BLOCK(1) 0.0936 ; 4 BOVKA (OCC1)
$OMEGA  BLOCK(1) SAME ; 5 BOVKA (OCC2)
;-------------------------------------------------------------
$SIGMA 1 FIX
;-------------------------------------------------------------
$ESTIMATION MAXEVAL=9999 METHOD=1 INTER
;-------------------------------------------------------------
```

# **S4. Pyrazinamide final model NONMEM control stream**

```
$PROBLEM    Method trial – pyrazinamide final model
$INPUT      ID AMT DV TIME EVID MDV OCC BLQ FFM_KG
$DATA       data.csv IGNORE=@
$ABBR DERIV2=NO
$SUBROUTINE ADVAN5 TRANS1
;-------------------------------------------------------------
$MODEL
NCOMPS=6 ;
COMP=(TRANSIT1,DEFDOSE)
COMP=(TRANSIT2)
COMP=(TRANSIT3)
COMP=(TRANSIT4)
COMP=(ABS)
COMP=(CENTRAL,DEFOBS)
;-------------------------------------------------------------
$PK
; ------- BSV
BSVCL   = ETA(1)
; ---------- BOV
BOVBIO = 0
IF (OCC.EQ.1)BOVBIO = ETA(2)
IF (OCC.EQ.2)BOVBIO = ETA(3)
BOVKA =  0
IF (OCC.EQ.1)BOVKA = ETA(4)
IF (OCC.EQ.2)BOVKA = ETA(5)
BOVMTT = 0
IF (OCC.EQ.1)BOVMTT = ETA(6)
IF (OCC.EQ.2)BOVMTT = ETA(7)
;------------------- Allometric scaling ----------------------
;   Body-size covariates
;   FFM_KG      : fat-free mass in kg
;-------------------------------------------------------------
TVFFM = 45; 45.6kg in METHOD TRIAL
;--------- Allometric scaling and covariates------------------
ALLMCL_FFM = (FFM_KG    / TVFFM)**0.75   ; CL ~ FFM^0.75
ALLMV_FFM  = (FFM_KG    / TVFFM)         ; V  ~ FFM^1.0
;----------------------------------------------------------
TVCL = THETA(1)*ALLMCL_FFM
TVV = THETA(2)*ALLMV_FFM
TVKA = THETA(3)
TVBIO = THETA(4)
TVMTT = THETA(7)
TVNN  = THETA(8)
;-----------Define parameters---------------------------------
CL  = TVCL*EXP(BSVCL)   ; CLEARANCE
V   = TVV)              ; CENTRAL VOL.
KA  = TVKA*EXP(BOVKA)   ; ABS. RATE CONSTANT
BIO = TVBIO*EXP(BOVBIO) ; BIOAVAILABILITY
MTT = TVMTT*EXP(BOVMTT) ; MEAN TRANSIT TIME (MTT)
NN  = TVNN              ; Number of transit compartments
;-------------------------------------------------------------
; re-parameterization
F1 		= BIO
KTR = (NN+1)/MTT
K12	=	KTR  ;Rate between transit CMT
K23	=	KTR   ;Rate between transit CMT
K34	=	KTR   ;Rate between transit CMT
K45	=	KTR   ;Rate between transit CMT
K56 =	KA
K60	=  CL/V
S6  = 	V ;CENETRAL COMPARTMENT SCALAR
;============================================================;
$ERROR
IPRED=A(6)/V
LLOQ = 0.2
CENS_THR = LLOQ
PROP = IPRED*THETA(5)
ADD = THETA(6)+(CENS_THR*0.2)

; BLQ handling method M7+, Wijk et al. CPT:PSP 2025 14(6)1042:1049
IF (ICALL/=4.AND.BLQ==1) ADD = ADD + LLOQ

W = SQRT(ADD**2+PROP**2)
IF (W.LE.0.000001) W=0.000001

IRES=DV-IPRED
IWRES=IRES/W

Y = IPRED + W*ERR(1)

;-------------------------------------------------------------
$THETA
(3.88) ; 1 CL [L/h]
(40.1) ; 2 V [L]
(3.45) ; 3 KA [1/h]
(1) FIX ; 4 BIO
(0.074) ; 5 PROP []
(0.895) ; 6 ADD [mg/L]
(0.424) ; 7 MTT
(4) FIX ; 8 NN

$OMEGA BLOCK(1) 0.0940 ; 1 BSV CL
;-------------------------------------------------------------
$OMEGA BLOCK(1) 0.0282 ; 2 BOVBIO (OCC1)
$OMEGA  BLOCK(1) SAME  ; 3 BOVBIO (OCC2)
;-------------------------------------------------------------
$OMEGA BLOCK(1) 0.653 ; 4 BOVKA (OCC1)
$OMEGA  BLOCK(1) SAME ; 5 BOVKA (OCC2)
;-------------------------------------------------------------
$OMEGA BLOCK(1) 2.10 ; 6 BOVMTT (OCC1)
$OMEGA  BLOCK(1) SAME ;  7 BOVMTT (OCC2)
;-------------------------------------------------------------
$SIGMA 1 FIX
;-------------------------------------------------------------
$ESTIMATION MAXEVAL=9999 METHOD=1 INTER
```
